# The asymmetric photosynthetic characteristics of the isobilateral sorghum leaves under the illumination of the diffuse light

**DOI:** 10.3389/fpls.2023.1218076

**Published:** 2023-07-13

**Authors:** Xiaolin Wang, Tao Wu, Muhammad Asim, Aifen Ling, Yanguo Sun, Yi Shi, Huifeng Yan

**Affiliations:** ^1^ Tobacco Research Institute, Chinese Academy of Agricultural Sciences, Qingdao, China; ^2^ Research and Development of Center, Liangshan Branch of Sichuan Tobacco Company, Xichang, China; ^3^ Center for Excellence in Molecular Plant Sciences, Chinese Academy of Sciences (CAS), Shanghai, China

**Keywords:** dorsoventral asymmetry, leaf structure and photosynthesis, stomatal conductance, diffuse light, *Sorghum bicolor L*.

## Abstract

The difference between photosynthesis on the two leaf sides (dorsoventral asymmetry) of photosynthesis is important for light-use patterns, but the asymmetry is environment dependent. Its role in photosynthetic regulation has been intensively studied, but little is known about the impacts of direct and diffuse light on the asymmetry. Because of the current changing fraction of diffuse light in sky radiation, this study investigated the dorsoventral asymmetry of photosynthetic traits under direct and diffuse light conditions in an important food and energy crop, *Sorghum bicolor L*. A unique method was used to investigate the specific gas exchange of each leaf surface. Anatomical and morphological traits were different between the two surfaces of sorghum leaves, which might result in photosynthetic asymmetry. The variations in photosynthetic rates and stomatal conductance were significant between the two surfaces in direct and diffuse light, but the degree of dorsoventral asymmetry decreased in diffuse light. The integrated *P*_N_ and *G*_s_ of the adaxial illumination were significantly higher than that of abaxial illumination both in direct and diffuse light in sorghum leaves, but the *ASI* of the integrated *P*_N_was 2.83 in direct light, while significantly dropped to 1.69 in diffuse light. Significant morphological differences between the two surfaces might cause photosynthetic asymmetry in the sorghum leaves. The variations of specific gas exchange were significant between direct and diffuse light, including in the incident and self-transmitted light. Compared with direct light, diffuse light reduced the stomatal sensitivity, with the degree of decline being greater in the adaxial surface, which caused weak dorsoventral asymmetry in photosynthesis. The specific photosynthetic characteristics in sorghum leaves varied obviously in direct and diffuse light, including in the incident and self-transmitted light, which contributed to the different overall gas exchange. Compared with direct light, the decline of stomatal sensitivity, which showed positive correlation with stomatal density, caused weakened dorsoventral asymmetry in photosynthesis in diffuse light. The findings provide new insights into dorsoventral asymmetry and the impact of diffuse light on photosynthesis in isobilateral leaves.

## Introduction

The solar radiation reaching leaf surfaces is the primary driver of leaf photosynthesis (Mercado et al., 2009; Strada and Unger, 2016). Alterations in sky conditions influence the properties of the incidental light-illuminating plants (Mercado et al., 2009; Cheng et al., 2015). Under clear sky conditions, sunlight arrives in beams at the earth’s surface, and leaves are illuminated from a single direction (Urban et al., 2012; Williams et al., 2014), while under cloudy sky conditions, nearly all of the incoming light is diffuse, with clouds, haze or fog scattering the light before it reaches the plants (Brodersen et al., 2008). As the variations in atmospheric aerosols increase, more diffuse light reaches the earth’s surface (Mercado et al., 2009; Oliveira et al., 2011) due to an increasing diffuse light index (the fraction of diffuse light in total radiation, *DI*) in sky radiation. For example, direct solar radiation decreased, and diffuse solar radiation increased in North China in the past five decades (1959 - 2016) (Feng et al., 2019). Does the increase in atmospheric aerosol change the photosynthetic traits of plants? This might directly depend on the photosynthetic responses of leaf light-use patterns to direct and diffuse light. Plants can use diffuse light more efficiently than direct light on the scales of an individual leaf, stand, canopy or crown, and biome (Gu et al., 2003; Knohl & Baldocchi, 2008; Mercado et al., 2009; Urban et al., 2012; Williams et al., 2014). Simulation models and eddy covariance methods revealed that the gross primary productivity of a canopy or individual plant tended to be greater under cloudy sky conditions, owing to the high proportion of diffuse light in the total irradiance (Mercado et al., 2009; Urban et al., 2012; Williams et al., 2014). Under field conditions, direct light illuminates the adaxial surfaces, while the abaxial surfaces receive diffuse light. Thus, the plant must acclimate to different light conditions by altering structures and photosynthetic functions.

Photosynthetic asymmetry, the photosynthetic variation between the incident light illuminating on the different surfaces, is important in determining the leaf light-use efficiency of two surfaces and adaptation to field light conditions. In general, leaves of C_3_ dicots show dorsoventral asymmetry in the palisade mesophyll tissue on the adaxial side and spongy mesophyll tissue on the abaxial side. These leaves are so-called bifacial leaves, and their photosynthetic structures and functions have been intensively studied (Kumagai et al., 2014; Wang et al., 2019; Wang et al., 2020; Xiong & Flexas, 2020; Wall, Shellie et al., 2022), revealing internal gradients in light intensity and photosynthetic activity within leaves (Gorton et al., 2010). But there was no agreement on whether the response of adaxial and abaxial was consistent. Recent reports showed that the dorsoventral asymmetry of photosynthetic traits of bifacial leaves was determined to be dependent on light intensity (Kumagai et al., 2014) and affected by direct and diffuse light (Kumagai et al., 2014; Wang et al., 2020). The explanation was mainly that the palisade tissue of bifacial leaves is the principal mesophyll cells, and the light reaching to palisade mesophyll is crucial to the photochemistry reaction (Wang et al., 2020; Xiong & Flexas, 2020). The difference in size, shape, and arrangement between palisade and spongy mesophyll cells changes the transmission path of the incident light. In the isobilateral leaves, the mesophyll of the sorghum leaves is not differentiated into spongy and palisade tissues, especially in the amphistomatic leaves (stomata on both surfaces), and dorsoventrally asymmetrical gas exchange was not obvious (Moss, 1964). However, different photosynthetic responses to adaxial and abaxial illumination have been observed in some C_4_ monocotyledonous plants (Long et al., 1989; Driscoll et al., 2006; Smith, 2008; Soares-Cordeiro et al., 2009; Soares-Cordeiro et al., 2011). The isobilateral leaves have obvious dorsoventral variations in photosynthetic functions between the adaxial and abaxial sides. Soares et al (2008) reported that abaxial contributions to leaf-specific photosynthesis were greater than adaxial contributions, independent of whether the adaxial or abaxial surface was illuminated with the same light intensity, owing to the greater number of stomata on the abaxial surfaces (Soares et al., 2008; Soares-Cordeiro et al., 2009). The tissue of these leaves is divided into two separate compartments by compact mesophyll tissue (Long et al., 1989; Smith, 2008; Soares-Cordeiro et al., 2009), and the air from stoma of one side could not diffuse to another side for Calvin cycle. Variations in photochemical activities involving light capture, reaction center activities, and electron transfer rates could also result in photosynthetic asymmetry between two surfaces, but related reports are limited (Xiong and Flexas, 2020).

Stomatal behavior dominates in photosynthetic variations before the non-stomatal limitation happens (Harrison et al., 2020). Most terrestrial plant species have hypostomatic leaves, or amphistomatic leaves with more stomata in abaxial surfaces. The location patterns of stomata was strongly associated with photosynthesis, this suggested that the distribution of stomata between the two surfaces provides an adaptive advantage under field conditions (Muir Christopher et al., 2014; Jordan et al., 2015; Richardson et al., 2017). The more evenly amphistomy allows leaves better to adapt high light and dry environment, and the amphistomy has also been associated with fast-growing species or herbaceousness (Muir, 2015). Most terrestrial plant leaves have to balance CO_2_ uptake and water loss through the stoma, especially in high light or dry environments, the amphistomatic leaves were discovered obvious advantage with the indispensable condition that the leaf can respond independently to a gradient in evaporative demand (Richardson et al., 2017; Wall et al., 2022). The shade leaves in sunflower similarly absorb direct and diffuse light, leading to a similar photosynthetic output regardless of light directionality (Earles et al., 2017). A main possible mechanism whereby diffuse light stimulates the photosynthesis of individual leaves involves sufficient stomatal openness and high stomatal conductance, which are caused by light properties and thermal effects (Reinhardt and Smith, 2008; Reinhardt et al., 2010; Urban et al., 2012; Li & Yang, 2015). The self-transmitted light is the light arriving at the unilluminated side (residual light intercepted by the illuminated side), and it could contribute a large proportion of whole-leaf photosynthesis (Wang et al., 2008). In sun-grown leaves of *H. annuus L*., the sensitivity of abaxial stomata to light might be greater than that of adaxial stomata, and the abaxial stomata might be more sensitive to the self-transmitted light than to direct light. The self-transmitted light varies between direct and diffuse light, forming up to 10% - 20% of the incident light intensity (Earles et al., 2017). Therefore, is stomatal behavior involved in dorsoventral asymmetry of photosynthetic traits in diffuse light compared to the direct light in the amphistomatic isobilateral leaves?

The sorghum (*Sorghum bicolor* L.) leaves are typical isobilateral leaves, mesophyll is often lengthened to appear as palisade-liked tissue. The microscopic morphology of the sorghum leaves used in this study showed physical restrictions in the dorsoventral airspace (Soares-Cordeiro et al., 2009; Jiang et al., 2011; Wang et al., 2019). Moreover, as a C_4_ plant, the CO_2_-fixation efficiency is greater than that of C_3_ plants, which causes a stronger correlation between the photosynthetic rate and intercellular CO_2_ concentration. We attempted to explain how the isobilateral leaves regulate photosynthesis under diverse direct and diffuse light conditions and tested the following hypotheses: (i) photosynthetic asymmetry is caused by different structures and light properties, and (ii) diffuse light weakens photosynthetic asymmetry by altering stomatal behavior. This study may specify the impact of increasingly diffuse light on crop yields in the future.

## Materials and methods

### Field sites and plant material

Experiments were conducted in an experiment field in Beijing (115.7°E–117.4°E, 39.4°N–41.6°N). The sorghum hybrid Liaoza 13 was selected for investigation. The seeds (provided by National Center for Sorghum Improvement, Liaoning province, PR China) were imbibed on wet paper for 1 d, and germinated seeds were sown in containers (30 cm ×20 cm × 10 cm) filled with vermiculite. Plants were watered every 2 days (d). Two weeks later, 4-leaf seedlings were transplanted into plastic pots (15 cm in diameter, 20 cm in height) containing Hoagland’s nutrient solution and grown in a site having simple rain shelters in the field exposing to natural light with a maximum intensity at 1800 ± 23 μmol photons m^-2^s^-1^. The nutrient solution was renewed every 3 d. The seedlings were used for measurements after 20 d.

### Measurement of leaf optical properties

Leaf reflectance and transmittance on each surface of the sorghum leaves were measured using a bifurcated fiber optic cable and a leaf clip (PP Systems, USA) across the spectrum of 310 - 1100 nm at 1-nm intervals. To calculate reflectance, leaf spectral radiance was divided by the radiance of a 99% reflective white reference standard (Spectralon, Labsphere, North Dutton, NH, USA) (Gamon and Surfus, 1999). Leaf transmittance was measured using two straight-fiber optics and a custom-made device. One straight-fiber optic irradiated the leaf from the adaxial side, and the other was used as a detector on the abaxial side. Absorptance = 1- reflectance - transmittance. The reflectance, transmittance, and absorptance could be obtained over 300 - 1100 nm wavelength. The average photosynthetically active radiation (PAR) (reflectance, transmittance, and absorptance of leaves was calculated from a trapezoidal integration over the 400 - 800 nm wavelength.

### Measurement of gas exchanges

**Figure 1:**
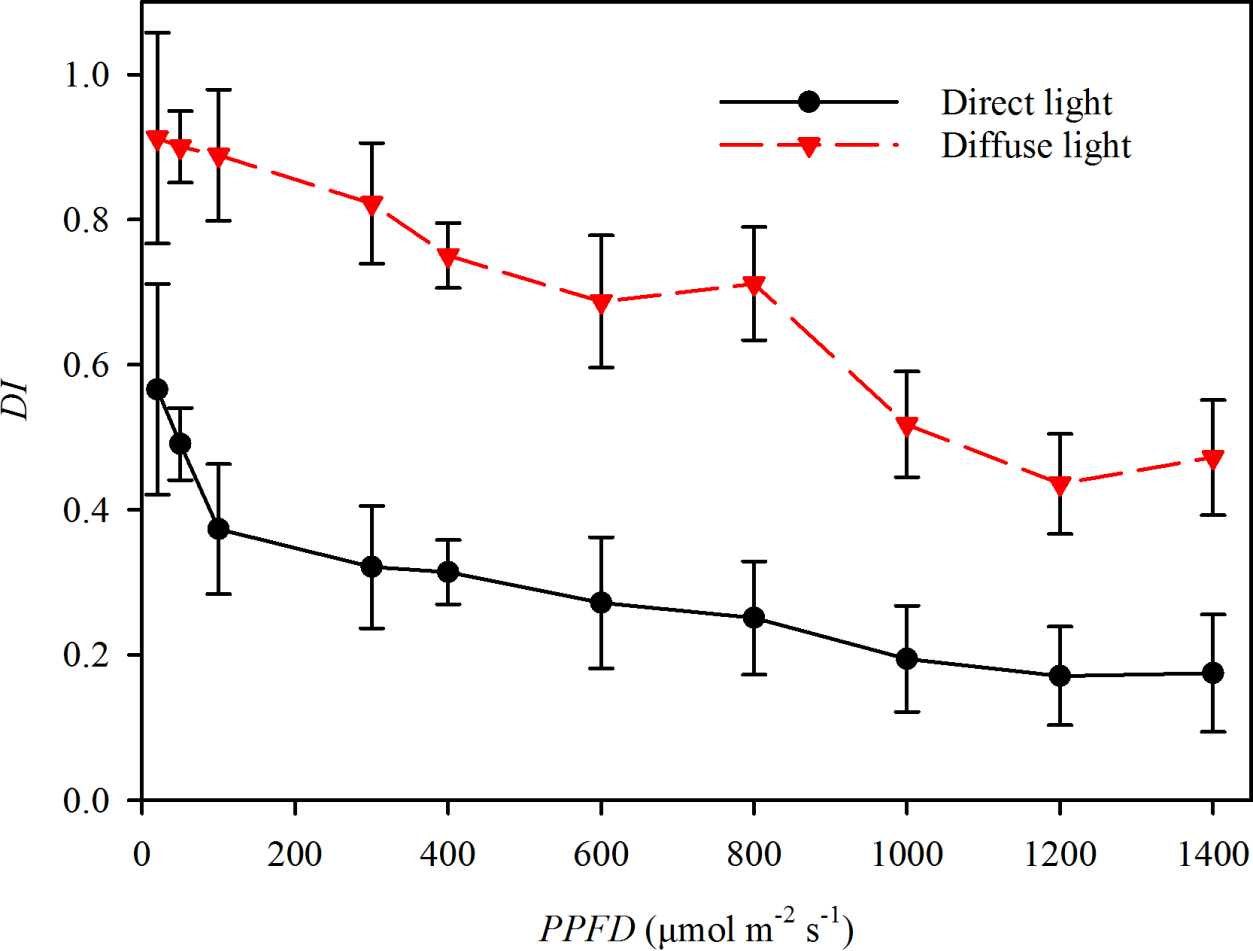
The diffuse index (*DI*) of the direct light (closed circles and solid line) and diffuse light (closed triangles and dotted line) in the experiments.

Photosynthetic gas exchange rates were measured using an infrared gas analyzer (Li-6400, Li-Cor, Lincoln, NE, USA) equipped with a combined LED light source which contained multispectral hybrid white light. By setting the voltage, the light source could provide direct light with an intensity range from 0 μmol m^-2^ s^-1^ to 1800 μmol m^-2^s^-1^. All the light near the earth’s surface is diffuse, which atmospheric aerosols have scattered. In many ecological studies, the *DI* mostly remained less than 0.27 during sunny days and more than 0.65 during cloudy days, respectively (Roderick et al., 2001; Li et al., 2004; Urban et al., 2007; Urban et al., 2012; Wang et al., 2016). This study measured the *DI* over the 400 to 800 nm wavelength range using a fiber optic spectrometer equipped with a cosine corrector (AvaSpec-ULS 2048XL, Avantes, Netherland). The light with *DI* > 0.55 was recognized as direct light from a LED, and the light with *DI* < 0.27 was recognized as diffuse light from using a scattered plate (Fotodiox Inc., USA) to convert the direct light to diffuse light (**Figure 1**).

A transparent film (TF) that passed our optical testing was used to block the flux between the leaf surface and chamber in a gas-exchange analyzer system (LI-6400), allowing the gas exchange rates of the opposite surfaces to be measured. Thus, the integrated overall and specific gas exchange rates of a leaf in direct, diffuse, and self-transmitted light were measured as follows:

(i) Adaxial specific gas exchange rates in direct light and diffuse light: the TF was placed between the lower chamber and the abaxial surface, and direct and diffuse light was illuminated on the adaxial surface;
(ii) Abaxial specific gas exchange rates in direct light and diffuse light: the TF was placed between the lower chamber and the adaxial surface, and direct and diffuse light was illuminated on the abaxial surface;
(iii) Adaxial specific gas exchange in self-transmitted light: the TF was placed between the upper chamber and the abaxial surface, and direct and diffuse light was illuminated on the abaxial surface;
(iv) Abaxial specific gas exchange in self-transmitted light: the TF was placed between the upper chamber and the adaxial surface, and direct and diffuse light was illuminated on the adaxial surface;
(v) The integrated gas exchange in direct and diffuse light: the incident light was illuminated independently on the adaxial and abaxial surfaces without the TF.

In total, 24 plants were selected for the studies. The plants were measured in batches for either integrated or specific measurements. All measurements were performed on the fully-expanded leaves. Measurements were conducted from 9:00 to 12:00 in a leaf chamber set at 30°C, with 45% - 50% relative humidity, an ambient CO_2_ concentration of 380 µmol mol^-1,^ and an irradiance of 1,000 μmol m^-2^ s^-1^. The light-response curves for gas exchange were investigated under direct, diffuse, and self-transmitted light, with the light intensity gradient of 1,200, 1,000, 800, 600, 400, 300, 250, 200, 150, 100, 50, 20, 0, 0 and 0 μmol m^-2^ s^-1^, three logging in 0 μmol m^-2^ s^-1^ was to obtain the steady gas-exchange in dark condition. Our preliminary study of leaf optical properties showed that the reflectance rates of either surface were 10%, and the absorbance rates of two surfaces were 85.4% under the LED light. We assumed that the absorbance rate of the two surfaces was the same, so the residual light reflected and intercepted by illuminating side was 47.7% of the incident light. Thus, the photosynthetic light-response curves under incident and self-transmitted light were obtained respectively by using the full and 47.5% of incident light intensity as the actual PPFD (*PPFD*_act_). The intercellular CO_2_ concentrations (*C_i_*) corresponded with the values of *P_N_* and *G_s_* in adaxial illumination and abaxial illumination, respectively. The ratios between the intercellular CO_2_ concentration (*C*_i_) and atmospheric (*C*_a_) CO_2_ concentration (*C*_i_ */C*_a_ ratios) were presented in separate experiments. When the leaf was acclimated in 1,200 μmol m^-2^ s^-1^ for 30 min, the CO_2_ supply in the chamber was controlled by a CO_2_ supply system equipped with a CO_2_ supplysystem equipped with LI-6400 to maintain the *C*_a_ near 310 µL L^-1^. The values were logged when *P*_N_ and stomatal conductance (*G_s_*) reached a steady state, and *C*_a_ reached 310 µL L^-1^. This study measured the boundary layer conductance with wet filter paper before the gas-exchange measurement. In the experiments, the boundary layer conductance at the flow rate of 500 μmol s^-1^ was 0.87 mol m^-2^ s^-1^ and 0.96 mol m^-2^ s^-1^ for the adaxial and abaxial half-chambers, respectively. The apparent quantum yield (*AQY*) was the initial slope of the regression curve of *PPFD*_act_ versus *P*_N_.

### Stomatal densities and sizes on leaf surfaces

The *SD* was determined using the “leaf imprints” (Coupe et al., 2006). A fast-drying nitrocellulose and ethyl acetate were smeared evenly on the leaf surfaces to obtain a replica of the leaf surfaces. The replicas were observed under a light microscope (Olympus BH-2; Olympus Optical Co. Ltd, Tokyo, Japan), and a digital camera was used to photograph the replicas. Samples were taken from the same area of the leaves used for the gas-exchange measurements. The *SD*s (the number of stomata mm^-2^) were determined by the numbers of stomata with the constant area. We measured the long axis of each stoma to determine the size because the short axes of stomata change depending on the degree of opening. In total, 100 marked leaves from 10 individual plants per treatment were selected for measurement.

### Determination of stomatal sensitivity

The light-response curves of stomatal conductance were used for calculating stomatal sensitivities. Stomatal conductance increased as the light intensity increased. Stomatal conductance had a strong linear correlation with light intensity at low-light intensities (< 200 μmol m^-2^ s^-1^). The regression line of stomatal conductance (y) versus light intensity (x) was:

y= *β* x + *b*,

where *β* represents the initial slope of the regression line at low-light intensities, and the value of b was the steady stomatal conductance in the dark conditions. The value of *β* could reflect the stomatal insensitivity to light intensity (Wang et al., 2008).

### Transverse semi-thin leaf sections for optical microscopy

Leaf sections (2 × 2 mm) were immersed in a fixative consisting of 1% (v/v) par-formaldehyde and 3% (v/v) glutaraldehyde in a 0.1 mM sodium phosphate buffer. The sections were subsequently washed, post-fixated, and dehydrated (Wang et al., 2019). Samples were embedded in Spur resin. Transverse semi-thin leaf sections (1 μm) were observed using a light microscope (Nikon-E800, Scientific Imaging Inc, USA).

The mesophyll thickness of two surfaces, the area of the sub-stomatal cavity per unit transection (*S*_a_ */S*), and the chloroplast surface areas exposed to intercellular airspace per unit leaf area (*S*_c_ */S*) were determined from the pictures. Moreover, chloroplast surface areas exposed to intercellular airspace (*S*_c_) and the contact area between the bundle sheath and mesophyll cells (*S*_b_) in transverse semi-thin leaf sections were determined by (Soares-Cordeiro et al., 2009; Wu et al., 2014) methods. *S_b_* was calculated according to the following specific steps: (i) assuming that mesophyll cells were spheroids, following Thain (Thain, 1983), the total cell surface area per unit volume of tissue is equal to the total length of the cell profile perimeter in a unit area of a tissue section; (ii) the lengths and the widths of cells can be easily measured, and the curvature correction factors (*F*) can be determined by using length/diameter ratios, and (iii) the contact area of the chloroplast facing the outer mesophyll cells was considered as

*S*_b_ = *L*_c_/*W* · *F*,

where *L*_c_ represents the lengths of chloroplasts facing the mesophyll cells, and *W* represents the width of the leaf cross-section (Wu et al., 2014). The value of *F* in mesophyll cells of sorghum leaves was 1.45, and the values of *W* were calculated using the pictures of transverse semi-thin leaf sections with Image J software.

### The degree of dorsoventral asymmetry

The asymmetry index (*ASI*) was used in our study to reflect the degree of dorsoventral asymmetry. The *ASI*s for morphological and anatomic structures, and gas-exchange parameters, were calculated as the ratio of the value of the adaxial side to that of the abaxial side. By definition, if a given parameter shows the same value for both sides, its *ASI* is 1.00. Thus, the greater deviation of *ASI* from 1.00 denotes a greater degree of *ASI*.

### Statistical analysis

Data were subjected to separate statistical analyses using parametric tests. Differences between means were analyzed using Student’s *t* - tests. Regression lines were obtained by the least squares method. Differences in the regression coefficient and in intercept were detected using an analysis of covariance. The multiple comparisons were assessed using Tukey’s test. Linear regression models were fitted to pairwise scatter plots to analyze the relationship between stomatal sensitivity β and SD for each individual surface. Data were analyzed using the Statistical Package for Social Sciences (SPSS, Version 18.0, for Windows). Correlations of linear regressions were calculated using Sigmaplot (version 14.0).

## Results

### Leaf optical properties

**Figure 2:**
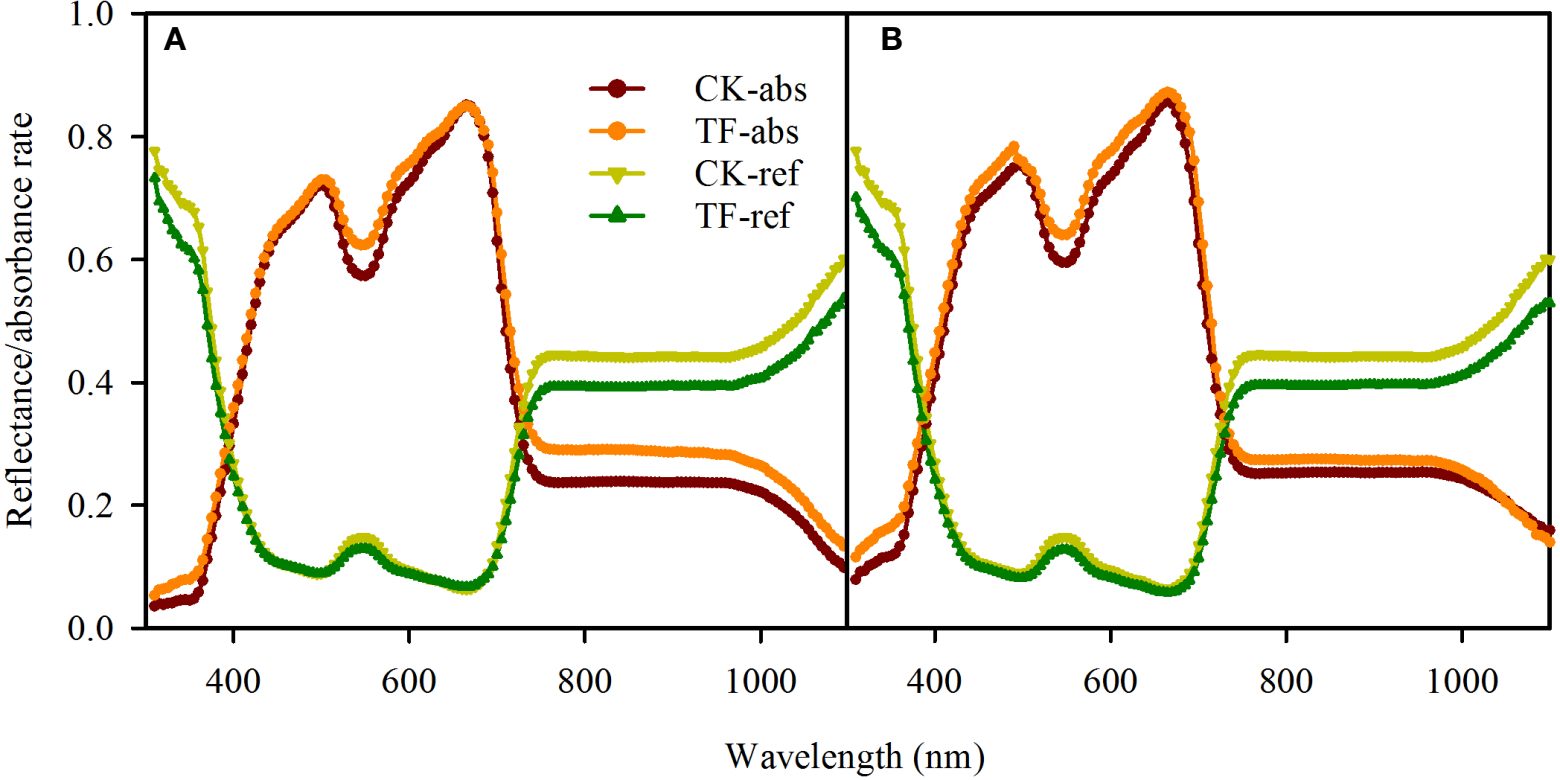
Effects of covering transparent film (TF) on the reflectance and absorbance rates (310-1100 nm) of adaxial surfaces **(A)** and abaxial surfaces **(B)** in sorghum leaves. TF-absorbance and TF-reflectance present respectively leaf absorbance and reflectance rate when the incident light arrived leaf surfaces through the TF, and CK-absorbance and CK-reflectance present respectively leaf absorbance and reflectance rate when the incident light arrived leaf surfaces without any coverage. Data are the mean ± SE of 50 leaves.

Our testing showed that the TF-covered treatment only changed the absorbance rate over 530 - 550 nm and 750 -1100 nm in adaxial illumination and 530 -550 nm and 590 – 620 nm in abaxial illumination (**Figure 2A**); the absorbance rate changed slightly (<5%) in rather a narrow band (**Figure 2B**). For this reason, it was considered that the leaf absorbance/reflectance rate in PAR (400 – 800 nm) was unchanged when covering the illuminated leaf surface with the TF.

**Figure 3:**
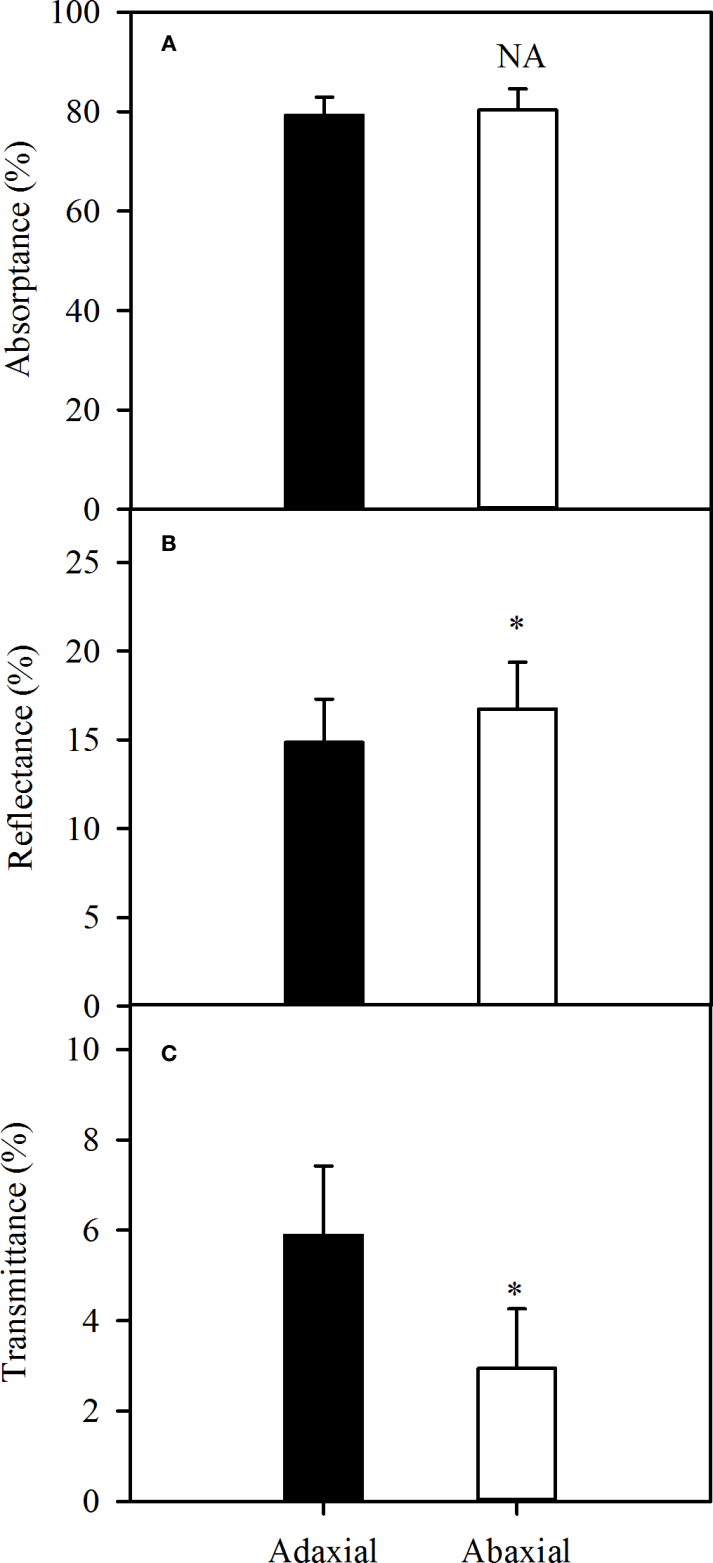
Variations of average leaf absorbance **(A)**, reflectance **(B)** and transmittance **(C)** over the wavelength of 400 - 800 nm in sorghum between adaxial surface (black bar) and abaxial surface (white bar). Data are the mean ± SE of 50 leaves. Bars superscripted by different letters are significantly different, based on one-way ANOVA (“*” for *P* = 0.05), NA, no significant between adaxial and abaxial values.

The absorbance could be up to approximately 80% of incident light, whether adaxial or abaxial illuminating over the photosynthetically active region of the spectrum (400 - 800 nm) (**Figure 3A**). Further, more light illuminated into the leaf as the result of lower reflectance in case of adaxial illuminating (P<0.05) (**Figure 3B**), but more light transmitted from the leaf (**Figure 3C**), which caused the no-significance of absorbance between two surfaces. Consequently, there was no difference in the absorbance levels between the two surfaces of sorghum leaves over the photosynthetically active wavelengths.

### Leaf morphology and structure

Transverse sections observed by light microscopy showed that the sorghum leaves were not differentiated into palisade and spongy tissues. However, the long axes of the adaxial mesophyll cells were longer than those of the abaxial mesophyll cells. There were many motor cells in the adaxial epidermis but none in the abaxial epidermis. The vascular bundles were surrounded by parenchymal cells, and the paths from bundle sheath cells to both surfaces were nearly identical. The sub-stomatal cavities of the adaxial and abaxial mesophyll cells were separated by compact mesophyll tissues (**Figure S1**). Both sides of leaves had stomata, and the *SD* of the abaxial surfaces were 1.5 times greater than those of the adaxial surfaces (*P* < 0.001), while the adaxial stomata lengths were approximately the same as the abaxial values (**Table 1**). Further analyses showed that the *S_a_/S*, *S_c_/S*, and *S_b_* of the abaxial surfaces were significantly greater than those of the adaxial surfaces (*P* < 0.01) (**Table 1**).

**Table 1:** Variations of stomatal density (*SD*), the long axis of stomatal aperture, the area of the sub-stomatal cavity per unit transection (*S*_a_ */S*), the chloroplast surface areas exposed to intercellular airspace per unit leaf area (*S*_c_ */S*) and the contact area between the bundle sheath and mesophyll cells (*S*_b_) between the adaxial and abaxial side in sorghum leaves.

	*SD* (number of stomata mm^-2^)	Long axis of stomatal aperture (μm)	*S*_a_ */S* (m^2^ m^-2^)	*S*_c_ */S* (m^2^ m^-2^)	*S*_b_ (m^2^ m^-2^)
Adaxial	78.6 ± 2.10	31.2 ± 2.31	0.85 ± 0.05	5.63 ± 1.11	2.12 ± 0.20
Abaxial	126.5 ± 3.30	30.3 ± 2.18	1.72 ± 0.06	9.45 ± 2.11	2.53 ± 0.30
N	108	120	20	20	20
P-value	<0.001	0.056	<0.01	<0.01	<0.01
ASI	0.62	1.02	0.49	0.60	0.84

All the parameters were calculated from samples with number of N in tables.

### Dorsoventral asymmetry of photosynthesis in sorghum leaves in direct and diffuse light

**Figure 4:**
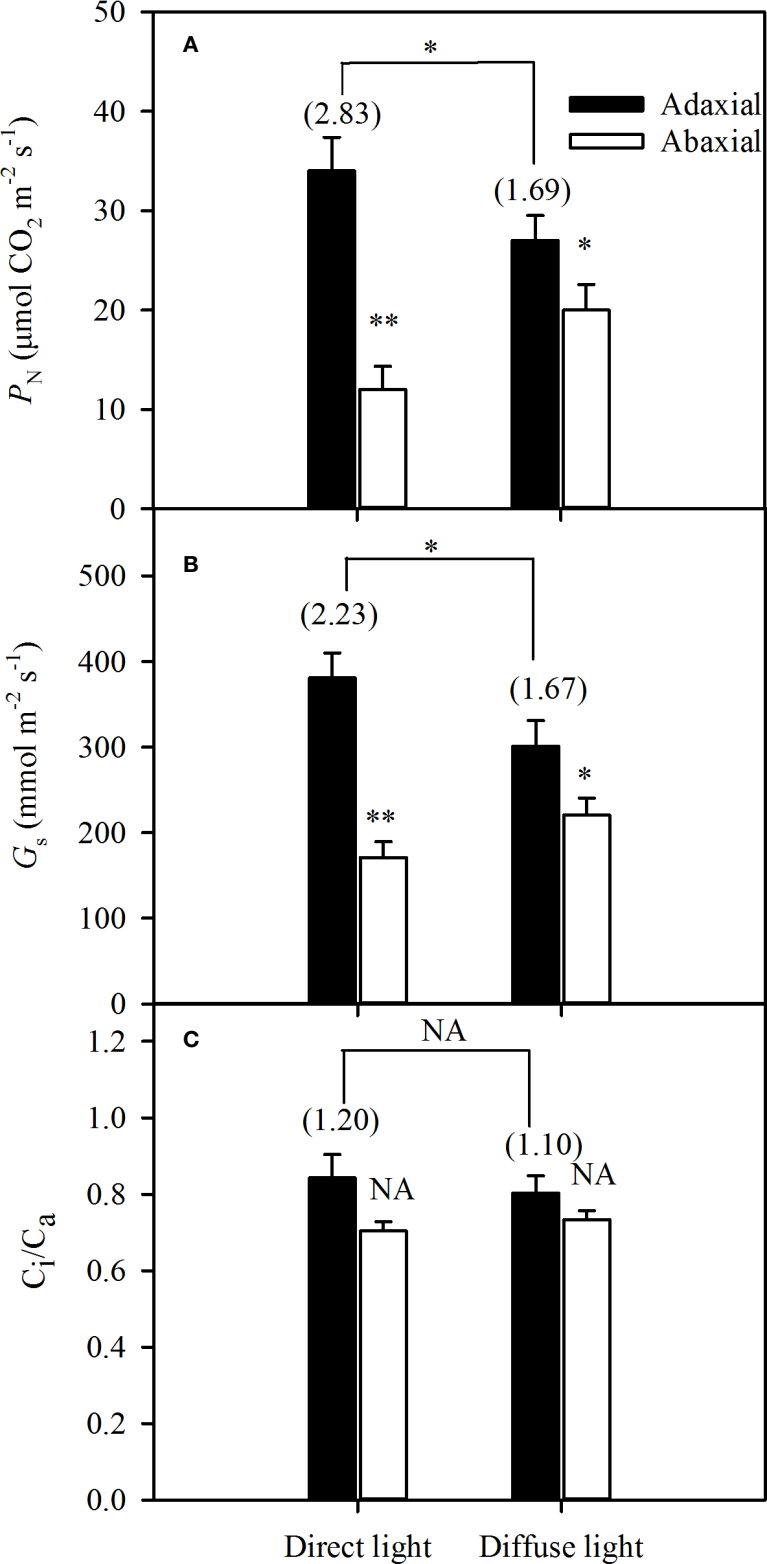
Variations of integrated gas exchanges of illuminating adaxial (black bars) and abaxial (white bars) leaf surfaces respectively in direct and diffuse light. **A** for photosynthetic rate (*P*_N_), **B** for stomatal conductance (*G*_s_) and **C** for ratio between the intercellular and the atmospheric CO_2_ concentrations (*C*_i_ */C*_a_). Values in parentheses are asymmetry index (*ASI*) of *P*_N_, *Gs*, and *C*_i_ */C*_a_, respectively. Data are the mean ± SE of 24 plants. Bars superscripted by different letters are significantly different, based on one-way ANOVA (“*” for *P* = 0.05, “**” for *P* = 0.01), NA, no significant between adaxial and abaxial values; the significance of *ASI* between direct light and diffuse light was shown on the transverse lines.

The integrated *P*_N_ and *G*_s_ of the adaxial illumination were significantly higher than that of abaxial illumination both in direct and diffuse light, but the *ASI* of the integrated *P*_N_was 2.83 in direct light, while significantly dropped to 1.69 (**Figure 4A**). In addition, the *G_s_* changed as the *P*_N_ varied, and the *ASI* of the integrated *G_s_* dropped from 2.23 in direct light to 1.67 in diffuse light (**Figure 4B**). The *C*_i_ */C*_a_ remained nearly constant under direct and diffuse light conditions, and the *G_s_* was not affected by the *C*_i_ (**Figure 4C**).

### The stomatal conductance and stomatal sensitivity of each side in direct and diffuse light

**Figure 5:**
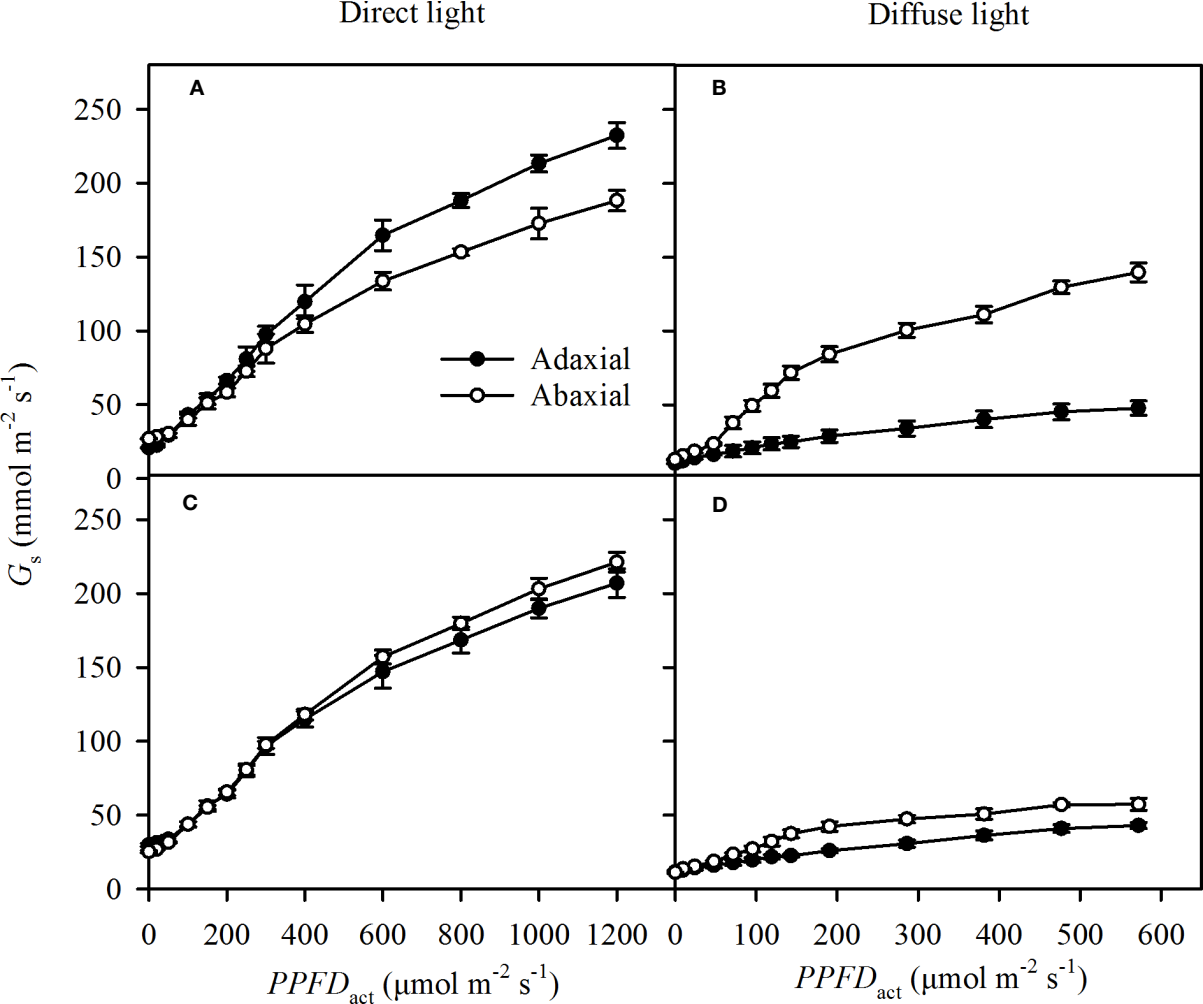
Light responses of the specific stomatal conductance (*G*_s_) in incident light (**A** for direct, **B** for diffuse) and self-transmitted light (**C** for direct, **D** for diffuse) when the light illuminating on adaxial (the closed circle) and abaxial (the open circle) surfaces, respectively. Data are the mean ± SE of 24 samples.

The specific *G_s_* increased rapidly as the light intensities (*PPFD*_act_) increased in direct light, including the direct incident light and self-transmitted in direct light (**Figures 5A, B**). The response of specific *G_s_* to *PPFD*_act_ was a little lower in diffuse light than that in direct light (**Figures 5C, D**) . The adaxial specific *G_s_* were greater than the abaxial specific *G_s_* at most *PPFD*_act_ in direct incident light (**Figures 5A**), while there was no obvious variation of the specific *G_s_* between adaxial and abaxial side in self-transmitted direct light (**Figures 5A, B**). The adaxial specific *G_s_* showed lower than the abaxial specific *G_s_* in diffuse light (**Figures 5B, D**).

The initial slope of the regression (*β*) reflected that the stomatal sensitivity. The *β* of adaxial surfaces was slightly greater (but significantly) than that of abaxial surfaces in direct incident light, while extremely lower than that of abaxial surfaces in self-transmitted in the direct light (**Table 2**). These results suggested that stomatal sensitivity varied significantly between the adaxial and abaxial surfaces when exposed to direct light, and these differences depended on which surface was illuminated. However, there was no significant variation of *β* value between the two surfaces whether in incident light or self-transmitted light when the leaves were exposed in diffuse light (**Table 2**), indicated that variation of the stomatal sensitivity between two leaf surfaces might be constant.

**Table 2:** The slope (*β*), Y-intercept (*b*) of regression lines of adaxial and abaxial surface in direct, diffuse, and self-transmitted light.

Light property	Leaf surface	*β*	b	P value	R^2^
Direct incident light	Adaxial	0.187b	29.41	<0.0001	0.973
	Abaxial	0.144d	32.37	<0.0001	0.970
Diffuse incident light	Adaxial	0.158c	35.62	<0.0001	0.972
	Abaxial	0.174bc	32.52	<0.0001	0.970
Self-transmitted in direct light	Adaxial	0.063e	27.38	0.0004	0.974
	Abaxial	0.230a	38.16	<0.0001	0.935
Self-transmitted in diffuse light	Adaxial	0.034f	26.58	0.0032	0.899
	Abaxial	0.032f	30.25	<0.0001	0.983

The determination ratio (R^2^), and P-value of ANOVA of regression equation y = *β* x + *b* are shown in the table. N=24. The statistical analysis (P < 0.01) compares data for *β*, the different letters representing significant differences of six cases from three light properties and two surfaces.

### Photosynthesis of each side in direct and diffuse light

**Figure 6:**
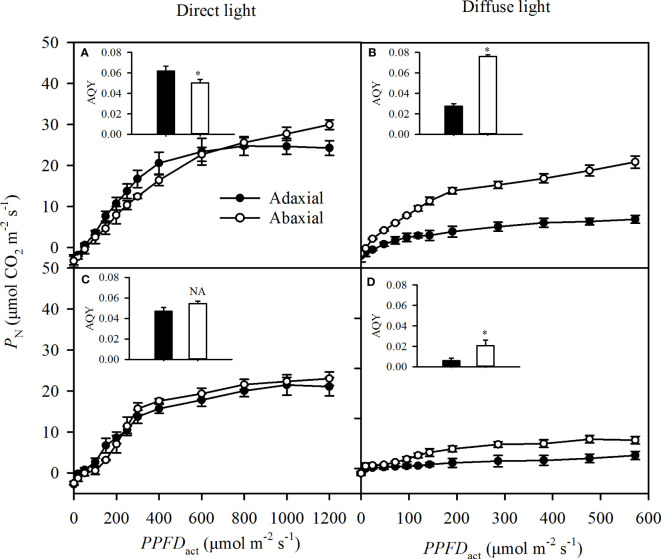
Light responses of the specific photosynthetic rate (*P*_N_) in incident light (**A** for direct, **B** or diffuse and self-transmitted light (**C** for direct, **D** for diffuse) when the light illuminating on adaxial (the closed circle) and abaxial (the open circle) surfaces, respectively. The inserted figure in each panel was the apparent quantum yield (*AQY*) of each treatment. Data are the mean ± SE of 24 samples. Bars superscripted by the "*" are significantly different between adaxial and abaxial values, based on one-way ANOVA, while NA for non-significant.

The adaxial specific *P*_N_ was greater than the abaxial specific *P*_N_ at the light below 600 μmol m^-2^ s^-1^, but lower than the abaxial specific *P*_N_ at the higher light intensity in direct light (**Figure 6A**), which was not consistent with the change of *G_s_* (**Figure 5A**). Changes of adaxial specific *P*_N_ as the *PPFD*_act_ increased were consistent with *G_s_* in diffuse and self-transmitted light (**Figures 6B–D**). The adaxial-specific *AQY* was significantly greater than the abaxial-specific *AQY* in direct light (**Figure 6A**) but was significantly lower in diffuse and two types of self-transmitted light (**Figures 6B–D**).

## Discussion

### Dorsoventral asymmetry of photosynthesis in direct or diffuse light

The number, size, and distribution of stomata are quite different in amphistomatic leaves, and amphistomaty has been associated with a greater capacity for gas diffusion because of the reduction in the pathway of the CO_2_ diffusion from the atmosphere to sites of carboxylation. Significant morphological differences between the two surfaces might cause photosynthetic asymmetry in the amphistomatic leaves (Wang et al., 2020). Field environments change rapidly, and photosynthetic regulation is required to adapt to diverse light conditions because leaf photosynthesis is optimized through evolutionary fine-tuning. In our investigation, in spite of the same intensity, the diffuse light decreased the integrated adaxial *P*_N_ and *G*_s_ compared with direct light, but in abaxial ones, the opposite happened (**Figures 4A, B**). However, this phenomenon could not reverse the dorsoventral asymmetry as the *ASI* value greater than 1 owing to >3.5 billion years of evolution in leaf photosynthesis.

Here, we examined the great dorsoventral asymmetry of both direct and diffuse light, and the *ASI* was greatly higher in direct light (2.83) than in diffuse light (1.69). Leaf morphology and structure affect the photosynthetic capacity through *SD*, stomatal size, and movement (Soares-Cordeiro et al., 2009), as well as mesophyll tissue (Oguchi et al., 2005; Earles et al., 2017) and vascular bundle (Oguchi et al., 2005; Soares et al., 2008) structures. Photosynthesis in C_4_ plants requires the collaboration of mesophyll cells and vascular bundle sheath cells. The gas exchange rate between these two types of cells plays a crucial role in photosynthesis (Sowinski et al., 2008). Here, we measured the *S_a_/S*, *S_c_/S*, and *S_b_* values between adaxial and abaxial mesophyll sides, which are important in determining *P*_N_. The greater *SD*, *S_a_/S*, *S_c_/S*, and *S_b_* values in the abaxial leaf side compared with the adaxial side demonstrated that the gas-supply potential was greater in the former than the latter (**Table 1**). The photosynthetic capacities of C_4_ isobilateral leaves exposed to direct light tend to be greater in abaxial surfaces, owing to the higher *SD* and higher *G*_s_ (Long et al., 1989; Driscoll et al., 2006; Soares et al., 2008; Soares-Cordeiro et al., 2011). Our results showed that the integrated adaxial *P*_N_ was higher than the abaxial one (**Figure 4A**), and the abaxial specific *P*_N_ was higher than or the same with adaxial one, respectively in direct (in high light) and diffuse light (**Figures 6A, B**). These findings could imply that self-transmitted light was of sufficient intensity to be effective for driving photosynthesis.

In addition to CO_2_-supply capacity, light-use capacity, which involves light absorptance and photochemical activity, is also a key trait in determining the *P*_N_. Leaf optical properties reflect the proportion of the light incident into leaves, which may be influenced by surface properties, pigment content, and leaf morphology (Gorton et al., 2010). However, our data demonstrated that leaf absorptance and reflectance profiles were almost the same on the adaxial and abaxial surfaces in sorghum over most of the 400-800 nm wavelength range (**Figure 3A**). This result was consistent with other studies on isobilateral leaves (Soares et al., 2008; Brodersen and Vogelmann, 2010; Gorton et al., 2010). In fact, the light environments of the two surfaces were more complex, whether illuminated on the adaxial or abaxial side. For example, the light that illuminated on the abaxial surface was rarely direct light but usually diffuse and self-transmitted light. In our experiments, the integrated *P*_N_ of illuminated the adaxial surfaces was greater than those of illuminated on abaxial leaf surfaces (**Figure 4A**), which was inconsistent with previous reports (Long et al., 1989; Driscoll et al., 2006; Soares et al., 2008; Soares-Cordeiro et al., 2011). Actually, the integrated *P*_N_ and *G*_s_, shown in **Figure 4**, was the sum of two surfaces, whether the incident light illuminated on the adaxial surfaces or the abaxial ones. For example, in direct light, the integrated *P*_N_ illuminating on the adaxial surface (the black bar in **Figure 4A**) was the integration of the adaxial specific *P*_N_ in direct light (**Figure 6A**, closed circle) and the abaxial specific *P*_N_ in self-transmitted light (**Figure 6C**, open circle), while the integrated *P*_N_ illuminating on the abaxial surface (the open bar in **Figure 4A**) was the integration of the abaxial specific *P*_N_ in direct light (**Figure 6A**, open circle), and adaxial specific *P*_N_ in self-transmitted light (**Figure 6C**, closed circle). Thus, we speculated that the greater photosynthesis on the adaxial illuminating (**Figure 4A**) was due to the higher adaxial specific *P*_N_ and *AQY* (**Figure 6A**) in direct incident light and slightly changed adaxial specific *P*_N_ and *AQY* in self-transmitted direct light (**Figure 6C**). Although the greater photosynthesis on the adaxial illuminating was observed than that on the abaxial illuminating (**Figure 4A**) in diffuse light, the *ASI* dropped from 2.83 to 1.69 compared with that in direct light. We can apply the same reasoning that the higher photosynthesis but reduced *ASI* (from 2.83 to 1.69) on the adaxial illuminating was due to significantly higher abaxial specific *P*_N_ and *AQY* in self-transmitted diffuse light and lower adaxial specific *P*_N_ and *AQY* in diffuse incident light. Thus, the apparent conflicts with photosynthetic asymmetry in previous conclusions (Long et al., 1989; Wang et al., 2008; Soares-Cordeiro et al., 2009; Urban et al., 2012; Cheng et al., 2015; Earles et al., 2017) might be caused undistinguished light properties. The results in **Figure 6** also showed that the adaxial specific *P*_N_ could be higher than abaxial one, indicated that symmetric photosynthetic characteristics of sorghum leaves might be changed by light intensity, which was consistent with the results in tobacco (Wang et al., 2020).

### Variations of stomatal sensitivity in direct and diffuse light

**Figure 7:**
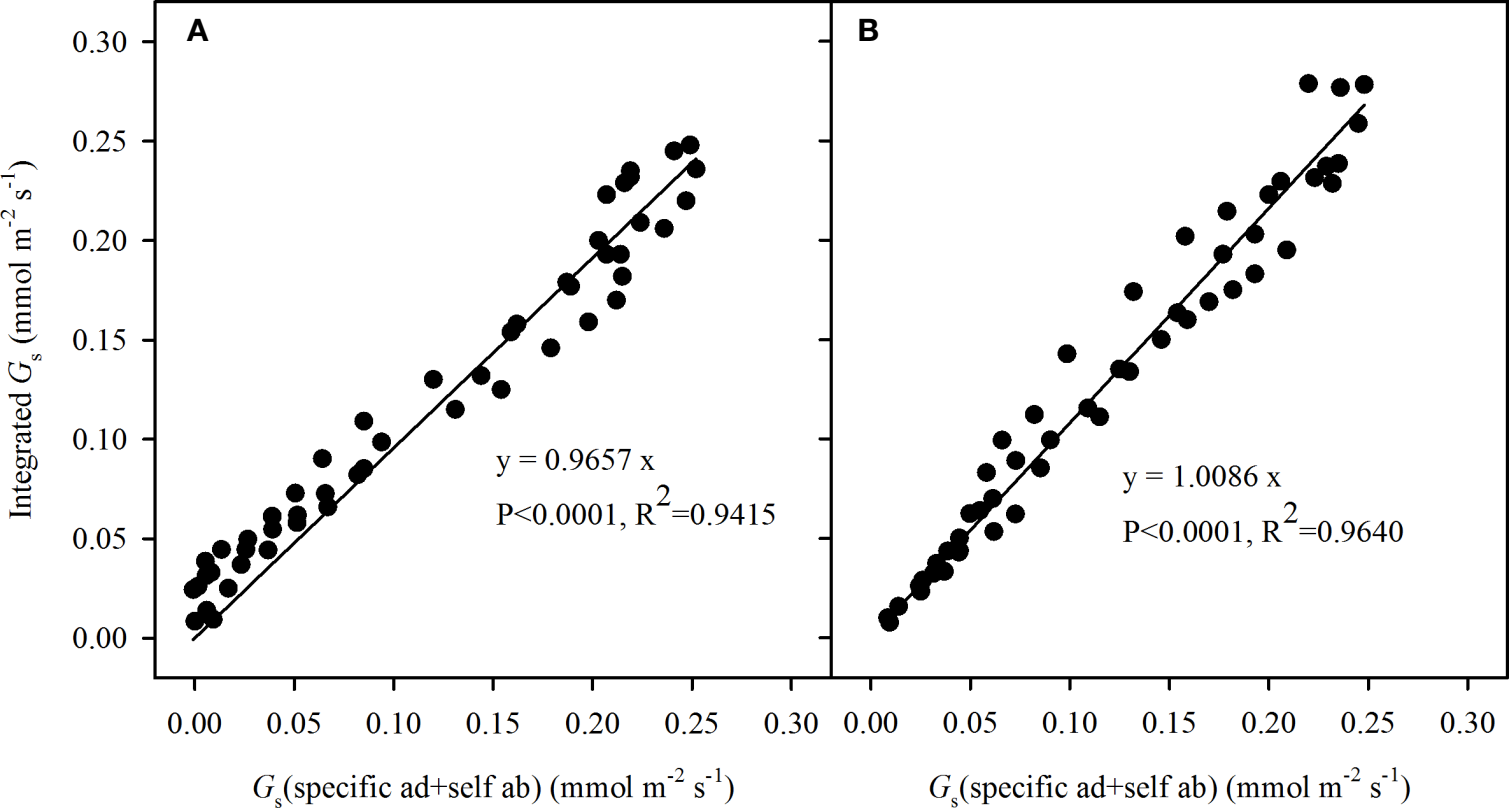
Correlations between the integrated stomatal conductance (*G*_s_) and the sum of adaxial specific *G*_s_ in incident light and abaxial specific *G*_s_ in self-transmitted light when the incident light illuminating on the adaxial **(A)** and abaxial **(B)** surfaces. The values were fitted by line regression (y = a * x), where a was the slope of the formula, the P values represented the significance of 1:1 test. N=90.

The CO_2_ diffusion was nearly unaffected by the opposite side, even though the stoma was blocked from the outside, owing to restriction between two sides by the compact mesophyll cells. This was further supported by the ratio of the integrated *G*_s_ to the sum of specific *G*_s_ responses shown in **Figure 7**, the calculated specific *G*_s_ in incident light + the *G*_s_ of the opposite side in self-transmitted light was closely corresponded with the integrated *G*_s_, respectively in adaxial illuminating (R^2^ = 0.9415, P < 0.0001, slope = 0.9657) and abaxial illuminating (R^2^ = 0.9640, P < 0.0001, slope = 1.0086), which was not significantly different from 1:1.

**Figure 8:**
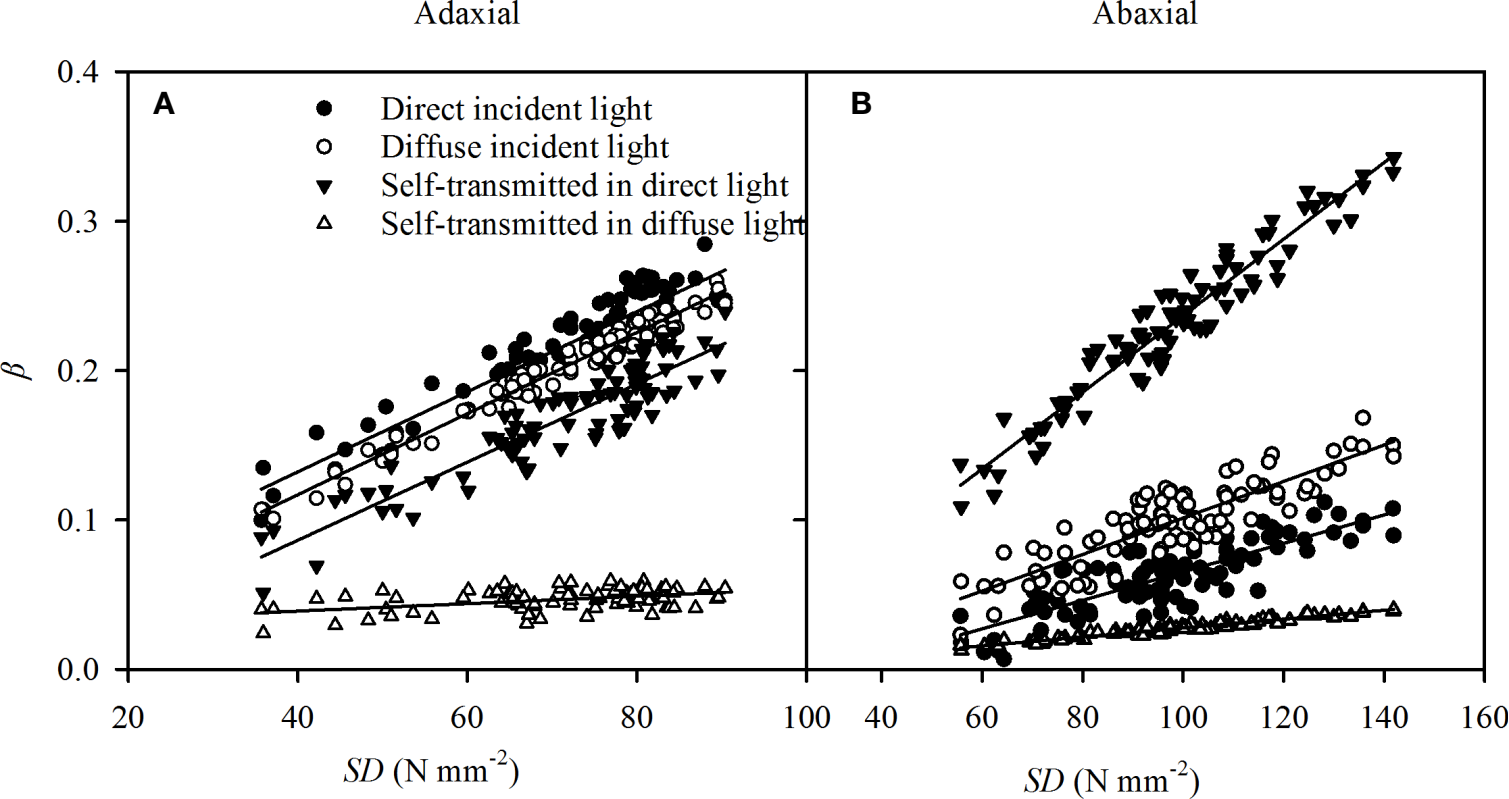
The correlations of stomatal sensitivity (*β*) in direct incident light (closed circles), diffuse incident light (open circles), and self-transmitted in direct light (closed triangles), self-transmitted in diffuse light (open triangles) with *SD* in adaxial **(A)** and abaxial **(B)** surfaces of sorghum leaves. The regression lines are as follows: y = 0.0028 x + 0.0251 (R^2^ = 0.864, *P* = 0.003) for adaxial *β*versus *SD* in the direct incident light, y = 0.0027 x + 0.084 (R^2^ = 0.971, *P* < 0.0001) for adaxial *β*versus *SD* in the diffuse incident light, y = 0.0026 x - 0.0181 (R^2^ = 0.841, *P* = 0.032) for adaxial *β* versus *SD* self-transmitted in direct light, y = 0.0002 x + 0.0291 (R^2^ = 0.2922, *P* < 0.0001) for adaxial *β* versus *SD* self-transmitted in diffuse light, y = 0.0010 x - 0.0305 (R^2^ = 0.699, *P* < 0.0001) for abaxial *β* versus *SD* in the direct incident light, y = 0.0012 x - 0.0212 (R^2^ = 0.756, *P* < 0.002) for abaxial *β*versus the diffuse incident light, y = 0.0025 x - 0.0182 (R^2^ = 0.901, *P* < 0.0001) for abaxial *β*versus *SD* self-transmitted in direct light, y = 0.0003 x - 0.0022 for abaxial *β* versus *SD* self-transmitted in diffuse light. N = 90.

As a major pathway of the gas exchange between the atmosphere and the plant, stomata plays an important role in the variation of adaxial and abaxial photosynthesis (Yu et al., 2004; Xu and Zhou, 2008). Light intensity arrived at the leaf surface is the most important ecological factor in stomata opening, despite the fact that stomata opening has been observed in the darkness in some species (Shimazaki et al., 2007). Although the *SD* was significantly greater in abaxial surfaces than in adaxial surfaces (**Table 1**), the abaxial specific *G*_s_ were lower than the adaxial specific *G*_s_ (**Figure 5A**) in the direct light. Stomatal sensitivity, as indicated by *β*, in leaf surfaces has an important role in *G*_s_(Goh et al., 2002; Wang et al., 2008), and the differences in the specific *β* between adaxial and abaxial surfaces were consistent with variations of *G*_s_ versus incident light (**Table 2**). The results in self-transmitted light were consistent with previous conclusions (Wang et al., 2008), but the specific *β* was greater in adaxial surfaces in direct light and nearly the same in the two surfaces in diffuse light (**Table 2**). Numerous *in vitro* investigations indicated that the stomatal sensitivity to light is greater on the abaxial surface than adaxial surface (Goh et al., 2002; Wang et al., 2008). The different conclusion might be due to the distribution of the stomatal apparatus (stomatal size and *SD*) on the two surfaces and the incident light, which might have considerable impacts on *β*. In our investigation, the stomata in both leaf surfaces were similar in size (**Table 1**), and *β* had strong correlations with *SD*s of adaxial (**Figure 8A**) and abaxial (**Figure 8B**) surfaces from different sorghum leaves. Hence, even though there was no direct effect of *SD* on specific *G*_s_, the specific *β* could be affected by the *SD*.

When the leaf was illuminated with polychromatic light, mesophyll cells of the illuminated side selectively absorbed red and blue light, while the remaining light that arrived at the opposite side consisted mostly of green light. Green light might be more efficient for stomata opening (Wang et al., 2008; Terashima et al., 2009). Wang et al. (2008) showed that the self-transmitted and leaf-transmitted light were more efficient in opening the abaxial stomata of sunflower leaves than the direct light, which was explained by the mostly green light in transmitted light (Wang et al., 2008; Falcioni et al., 2017). Consequently, discrepancies in the specific *β* in self-transmitted light might be cased variations in the spectral composition of incident light. Estimations of leaf stomatal sensitivities should take leaf surface traits and the light spectrum into consideration.

#### Regulation of dorsoventral asymmetry in sorghum leaves

The leaf thickness and specific leaf weight are highly correlated with photosynthetic capacity (Poorter et al., 2009; John et al., 2017). For an isobilateral leaf, the palisade-liked tissue of both sides increase light retention and absorption. Although our results showed that the *P*_N_s of adaxial illuminating were consistently higher than those under abaxial illumination both in direct and diffuse light (**Figure 4**), it’s important to emphasize the significant contribution of the abaxial side to overall leaf gas exchange. This finding aligns with previous reports in studies on wheat leaves (Wall et al., 2022). In isobilateral leaves, shaded adaxial mesophyll tissues decrease the probability of photoinhibition occurring in the abaxial side, and the greater photosynthetic rate of the abaxial side in a higher level of self-transmitted light could compensate for the loss of CO_2_ assimilation owing to stomata closure on the adaxial surface when exposed to extremely high radiation. Thus, the whole isobilateral leaf can maintain a high level of CO_2_ assimilation under excessive light, which has been reported previously (Long et al., 1989; Smith, 2008; Jiang et al., 2011). The greater adaxial G_s_ will not only increased CO_2_ uptake, but could also be critical in effective evapotranspiration for leaf cooling, to maintain optimal leaf temperatures for photosynthetic processes in high light conditions. Further studies are required to determine the roles of the differences stomatal behavior between the two surfaces, their significance in terms of evaporative cooling of the leaf in direct and diffuse light, respectively. In open field conditions, the diffuse light surrounding abaxial surfaces is much less than the direct light illuminating the adaxial surfaces (Wang et al., 2008; Gorton et al., 2010; Williams et al., 2014; Wang et al., 2016; Wang et al., 2021). Here, the greatest *P*_N_ occurred in adaxial surfaces illuminated by direct light, and the lowest *P*_N_ occurred in abaxial surfaces illuminated by direct light (**Figure 5A**). Thus, we hypothesize that the adaxial surfaces might mainly use direct light, while the abaxial surfaces might mainly use self-transmitted light, but not diffuse light, for photosynthesis under clear sky conditions. However, under cloudy sky conditions, the diffuse light surrounding adaxial and abaxial surfaces was approximately equal, although the light could be affected by planting density and cloud cover. The degree of asymmetry of *P*_N_ showed a decrease in diffuse light, which was consistent with the cloudy sky conditions.

## Conclusion

The specific photosynthetic characteristics in sorghum leaves varied obviously in direct and diffuse light, including in the incident and self-transmitted light, which contributed to the different overall gas exchange. Compared with direct light, the decline of stomatal sensitivity, which showed positive correlation with *SD*, caused weak dorsoventral asymmetry in photosynthesis in diffuse light. The photosynthetic asymmetry in sorghum leaves adapts to extreme light radiation and the changing light properties of different sky conditions, which could target plant breeding to increase productivity in the increasingly diffuse light of the future.

## Data availability statement

The raw data supporting the conclusions of this article will be made available by the authors, without undue reservation.

## Author contributions

XW did most of the data collection, XW, TW, YS, HY, YGS, and AL wrote the first draft and MA and HY edited and revised it. All authors contributed to the article and approved the submitted version.
