# Supplementary material for: The asymmetric photosynthetic characteristics of the isobilateral sorghum leaves under the illumination of the diffuse light
Source: Front Plant Sci. 2023 Jul 13;14:1218076. doi: 10.3389/fpls.2023.1218076 (PMC10374316; doi:10.3389/fpls.2023.1218076)
Supplement: Supplementary file 1 [file DataSheet_1.pdf]

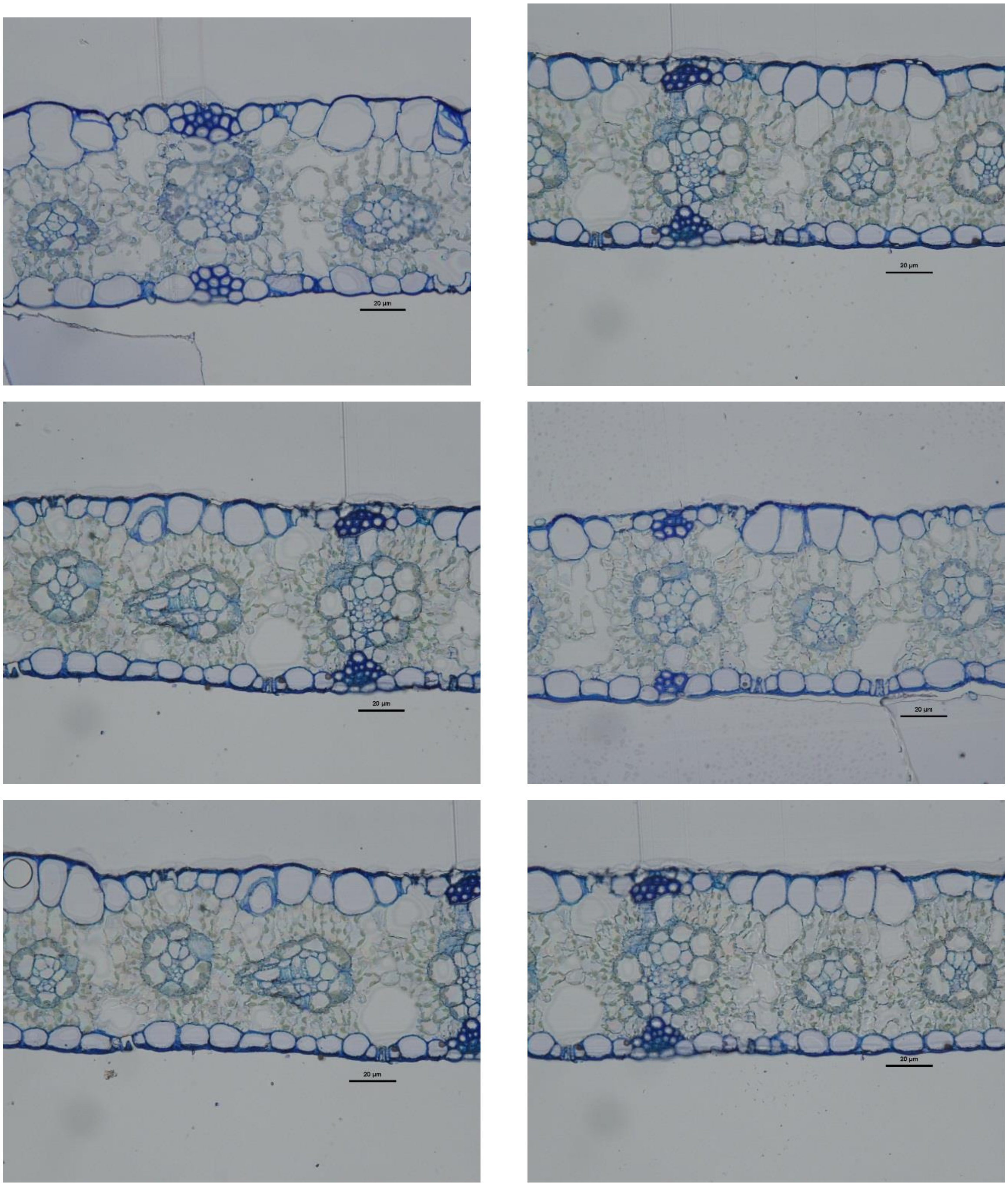
20 µm
20 µm
20 µm
20 µm
20 µm
20 µm

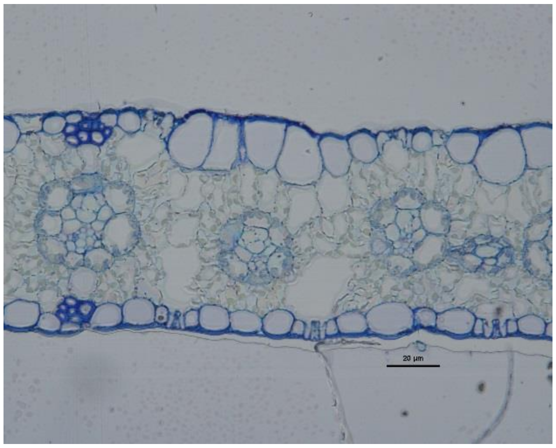
20 µm

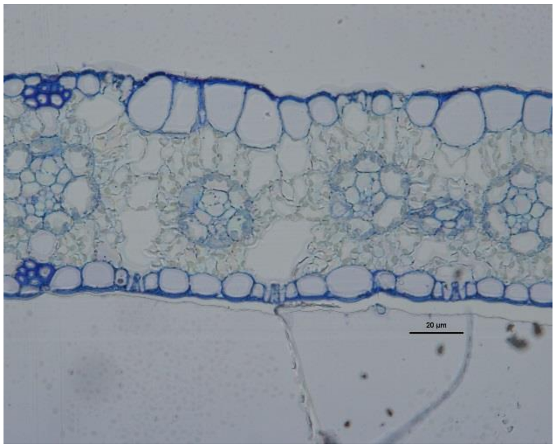
20 µm

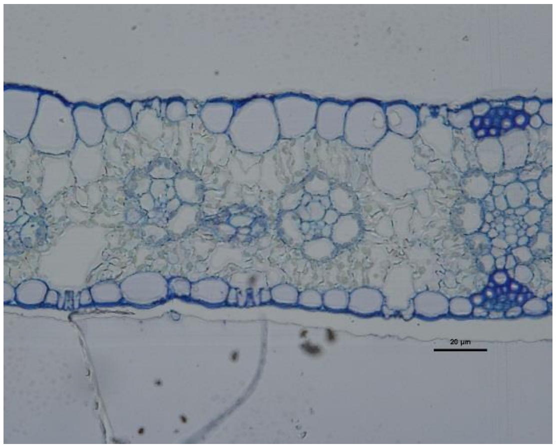
20 µm

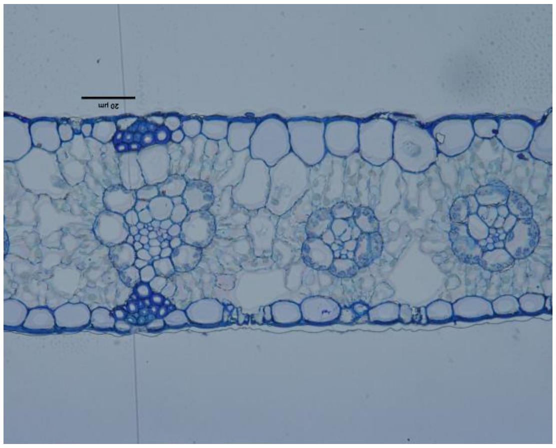

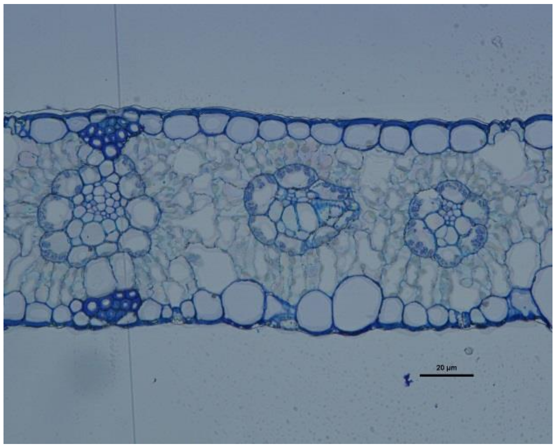
20 µm

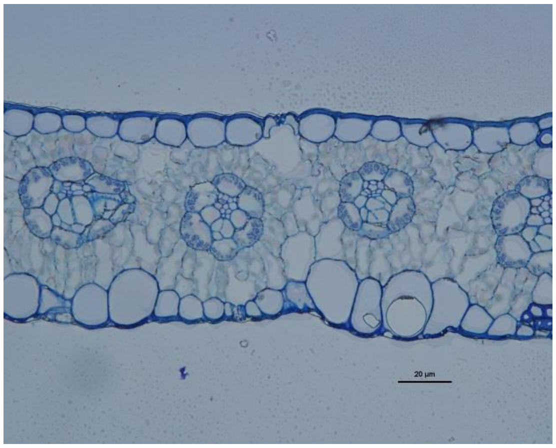
20 µm

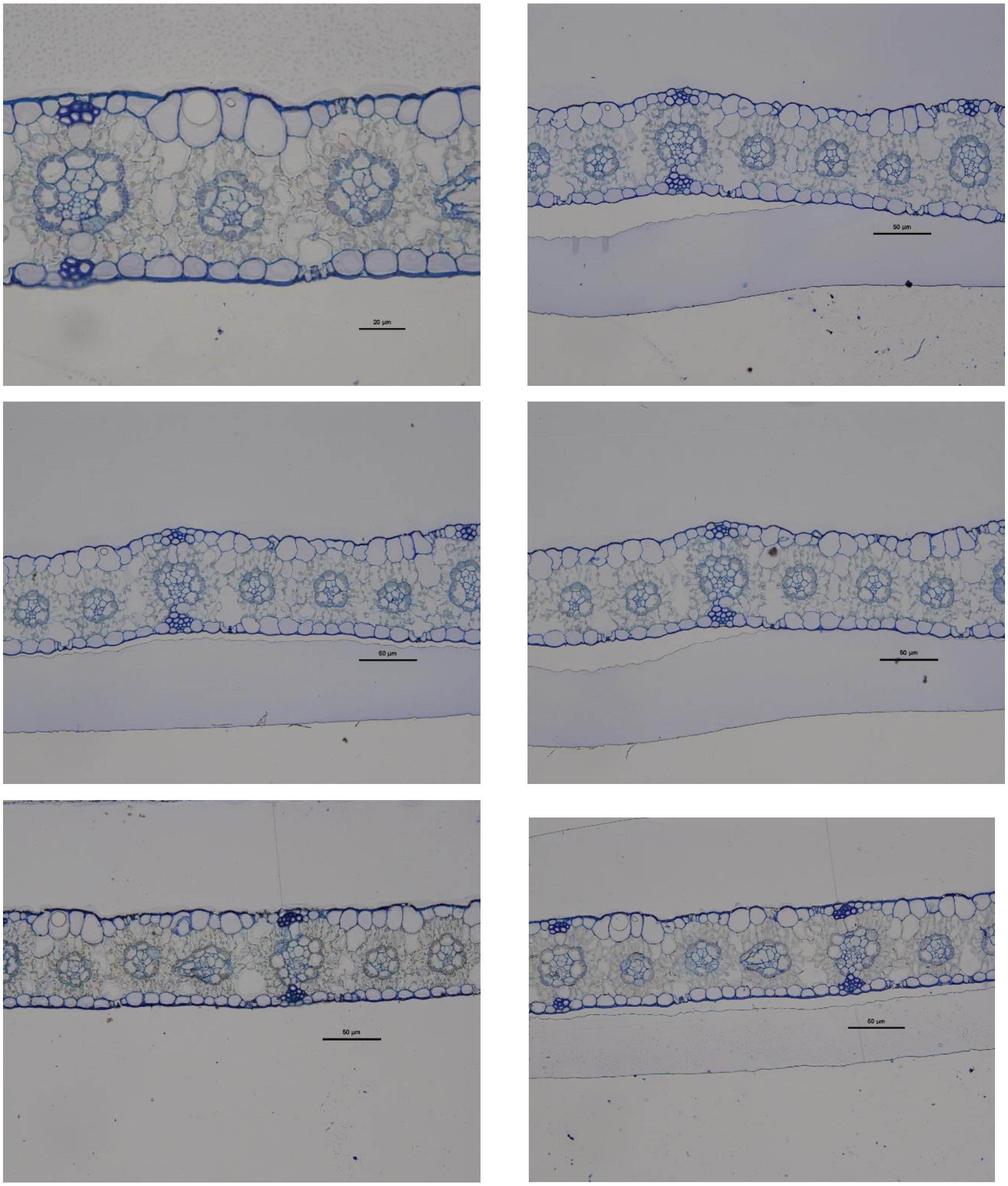

Fig. S1 The photos of transverse section of sorghum leaves.

The photos of transverse sections showed that the sorghum leaves were not differentiated into palisade and spongy tissues. However, the long axes of the adaxial mesophyll cells were longer than those of the abaxial mesophyll cells. There were many motor cells in the adaxial epidermis but none in the abaxial epidermis. The vascular bundles were surrounded by parenchymal cells, and the paths from bundle sheath cells to both surfaces were nearly the same. The sub-stomatal cavities of the adaxial and abaxial mesophyll cells were separated by compact mesophyll tissues.
